# Known and Unknown Transboundary Infectious Diseases as Hybrid Threats

**DOI:** 10.3389/fpubh.2021.668062

**Published:** 2021-07-14

**Authors:** Willy A. Valdivia-Granda

**Affiliations:** Orion Integrated Biosciences Inc., Manhattan, KS, United States

**Keywords:** biosurveillance, biosecurity, analytics and data mining, one health, risk

## Abstract

The pathogenicity, transmissibility, environmental stability, and potential for genetic manipulation make microbes hybrid threats that could blur the distinction between peace and war. These agents can fall below the detection, attribution, and response capabilities of a nation and seriously affect their health, trade, and security. A framework that could enhance horizon scanning regarding the potential risk of microbes used as hybrid threats requires not only accurately discriminating known and unknown pathogens but building novel scenarios to deploy mitigation strategies. This demands the transition of analyst-based biosurveillance tracking a narrow set of pathogens toward an autonomous biosurveillance enterprise capable of processing vast data streams beyond human cognitive capabilities. Autonomous surveillance systems must gather, integrate, analyze, and visualize billions of data points from different and unrelated sources. Machine learning and artificial intelligence algorithms can contextualize capability information for different stakeholders at different levels of resolution: strategic and tactical. This document provides a discussion of the use of microorganisms as hybrid threats and considerations to quantitatively estimate their risk to ensure societal awareness, preparedness, mitigation, and resilience.

## Introduction

Known and unknown transboundary infectious diseases that can affect humans, animals, and plants continue to emerge, reemerge, and persist in different locations worldwide (1, 2). The current pandemic of SARS-CoV-2 (3), the spread of the African Swine Fever Virus (ASFV) (4), and the impact of rice and wheat blast (5–7) are startling examples of how infectious diseases can become global challenges disrupting health, trade, and security. In addition to environmental factors, human activity contributes to the increasing emergence and reemergence of pathogenic microorganisms (8). Travel and trade are associated with 61% of the infectious disease outbreaks, and public health system failure and sociodemographic factors are accountable for 21 and 18% of these incidents (1). With the increasing number of travelers and the transcontinental movement of commodities, tracing the origins of natural or intentional pathogen introductions as acts of warfare or terrorism is difficult (9–11).

A perpetrator can take advantage of the unknown diversity of microbes, DNA genetic manipulation, and artificial intelligence to generate combinatorial organisms with new biological properties engineered to inflict harm (10). Denial and deception activities can cover offensive developments and transfer biological agents within state and non-state actors (8, 10, 12, 13). In addition to the direct threat posed to public health, the intentional introduction of infectious agents can cause political and economic destabilization, the coercion of markets, resources, and technology (14). Such an event could trigger trade restrictions or force countries to adopt quarantine policies detrimental to their overall interests. The offensive use of microorganisms targeting vulnerable soft targets can undermine the political and military response of countries affected (15). Because of the technological complexity of the attack, it could take years for the intelligence community to understand this biothreat terrain and more years for the research community to develop countermeasures against them.

Hybrid warfare is an active strategy that uses a combination of hybrid threats, including regular, irregular, terrorist, and criminal acts, against the most vulnerable sectors of a nation to achieve military and political goals. Because hybrid threats aim to destabilize and undermine societies, microbes are ideal agents to be used within a range of modes for sabotaging and attacking soft targets such as public health systems, agricultural production, and the food supply. Counteracting these hybrid threats requires a highly adaptable and resilient response. However, a fundamental dilemma of microbes as hybrid threats is whether to do something about them or if such hostile activity can be tolerated or absorbed (16). Another obstacle to thinking clearly about infectious diseases as hybrid threats is terminology (15, 17). Terms such as “hybrid” with the words “threats,” “warfare,” “activity,” “operations,” and “tactics” are common in military literature (16). Concepts such as “gray zone warfare,” “competition short of war,” and “modern political warfare” are conflated in policy publications (15, 18). However, these concepts are seldomly used in the biological sciences. This limited scope restricts possible scenario analysis of events where pathogens outbreaks emerged into terrestrial and aquatic environments, causing deaths, economic damage, and trade restrictions (19–21). This document introduces concepts and plausible scenarios about known and unknown microbes as hybrid threats. It also proposes enhancing current analyst-based biosurveillance, using improved capability assessment tools and implementing autonomous systems tracking infectious disease outbreaks in humans, animals, and plants.

## Transboundary Infectious Diseases as Hybrid Threats

In 2005, Mattis et al. stated that “Our conventional superiority creates a compelling logic for states and non-state actors to move out of the traditional mode of war and seek some niche capability or some unexpected combination of technologies and tactics to gain an advantage.” (22). Although the term “hybrid warfare” appeared along with irregular and asymmetric warfare, hybrid warfare rose to prominence in academic literature around 2014 to describe a change in the character of conflict (23). Under this assessment, adversaries combine conventional, unconventional, and irregular approaches with non-military means to neutralize superior conventional military power. Hybrid threats simultaneously and adaptively combine a wide range of violent and non-violent means to target vulnerabilities across society to undermine its functioning, unity, or will (15). The magnitude of the agricultural system and the food supply and its impact on public health and the economy make these value chains ideal targets for disruption. Through proxies and ambiguities, state-sponsored groups or self-funded attackers can employ microbes as hybrids threats. These agents can prevent or trigger early warning and avoid attribution, prosecution, or retaliatory responses.

Natural and human-made pathogens and toxins can be used as hybrid threats against soft targets as unknown unknowns (e.g., biothreats we are not even aware that we are unaware of) or known unknowns (e.g., biothreats beyond traditional biological agents). Artificial intelligence techniques simulating *in silico* “genomic rewinding (reversion) or forwarding” (adaptation) populations can lead to the synthesis of unknown ancestors or variants targeting specific ethnic groups (24), animal or plant breeds (25). These new unknown variants could not only defeat DNA synthesis screening, diagnostics, and other available countermeasures, including detection and antimicrobials, but could be more virulent and overcome immune profiles generated by vaccination or prior exposure to closely related pathogens. Computational biology can also derive more thermostable proteins modulating hyperthermic incubation and fever (26). Pathogens can be introduced in asymptomatic and diseased cold- or warm-blooded species and plants as a threat multiplier to produce the loss of lives of humans, animals and crops, triggering food product shortfalls, travel and trade restrictions, price spikes, and market distortions. These systemic failures can affect the capability of a nation and cause severe or catastrophic events (27).

## Limitations of Biosurveillance and Capability Assessment Systems

Internet-based communicable disease outbreak monitoring began to be implemented in the early nineties, but their deployment expanded significantly after the 2001 anthrax attacks in the US (28–32). Despite the progress, biosurveillance remains retrospective and suffers from tradeoffs among sensitivity, accuracy, and timeliness. Underreporting or misreporting infectious disease outbreaks depends on the varying degrees of detection capability, economic pressure, and trade dynamics of each country. Current reporting methods can lag by days or weeks in reporting the emergence of a narrow set of pathogens. The quality of this information can be affected by the degree of access, source moderation, language translation capacity, and the use of rule-based tools to discard potential noise (29, 33–37). The quality of information is often uncertain, leading to judgment errors in the products that support decision-making about prevention, preparedness, and response (38–42). Some biosurveillance tools inaccurately reported or underestimated the emergence or reemergence of infectious diseases, including Ebola, Zika, and Chikungunya, yellow fever, cholera, and more recently SARS-CoV-2 (29, 36, 43–46).

Pathogens can inflict harm depending on a complex set of social, economic, and preparedness parameters intrinsic to the public health, agricultural, and food supply chains of each nation (8). Therefore, an essential aspect of monitoring and tracking potential disruptions is assessing the mitigation capability of a nation. The logical framework dates back centuries from examining how military forces could defend against foreign and domestic adversaries. For the last 60 years, Military Balance has used quantitative criteria to evaluate the military potential of the state (47). By establishing capability indexes, military commanders and state managers evaluated how specific policies and modernization strategies improved readiness, efficiency, and sustainability. Following a similar approach, in 1990, the United Nations published the Human Development Report, which quantitatively ranked the health, education, and income of the nation in what is known as the Human Development Index (HDI) (48). Analysis of the HDI led to expanding this approach to quantify public health capabilities (33–35), societal safety, resilience, technological development and establish national strategies implementing joint activities performed by diverse government agencies and organizations, providing a unified solution to a problem or issue.

Public health indexes measuring the risk of catastrophic events to a country draw information from internationally accepted sources, and governments are crucial for global policy development (49–51). These indexes estimate the risk to infectious microorganisms by characterizing factors influencing vulnerability: demographic, health care, public health, disease dynamics, political-domestic, political-international, and economics. These evaluations assume that data aggregation and various statistics can explain the health differences and technological expertise and physical investments that correlate with technical capabilities (52). However, some of the components of these indexes are aggregated based on a score conversion, and clustering or classifying this data does not necessarily coincide with the ranking of the index. This situation arises because health indexes use quantitative indicators from semi-structured interviews or surveys that might be too subjective due to all the assumptions needed to build them (41, 53). Some estimations are biased by the nature of self-evaluation of each country or by the conclusions of a small number of researchers with restricted analytic and data mining tools. While global health indexes focus on the technical soundness of estimation methods, country users are more concerned about the extent of their involvement in the estimation process (54). Therefore, disparities in analytical tools used for near real-time infectious disease awareness vary across and within countries (54). As a result, available data may not be comparable over time, and estimates driven by covariates make scoring and interpretation difficult (39).

## Mitigating Hybrid Threats With Autonomous Biosurveillance

**Figure 1:**
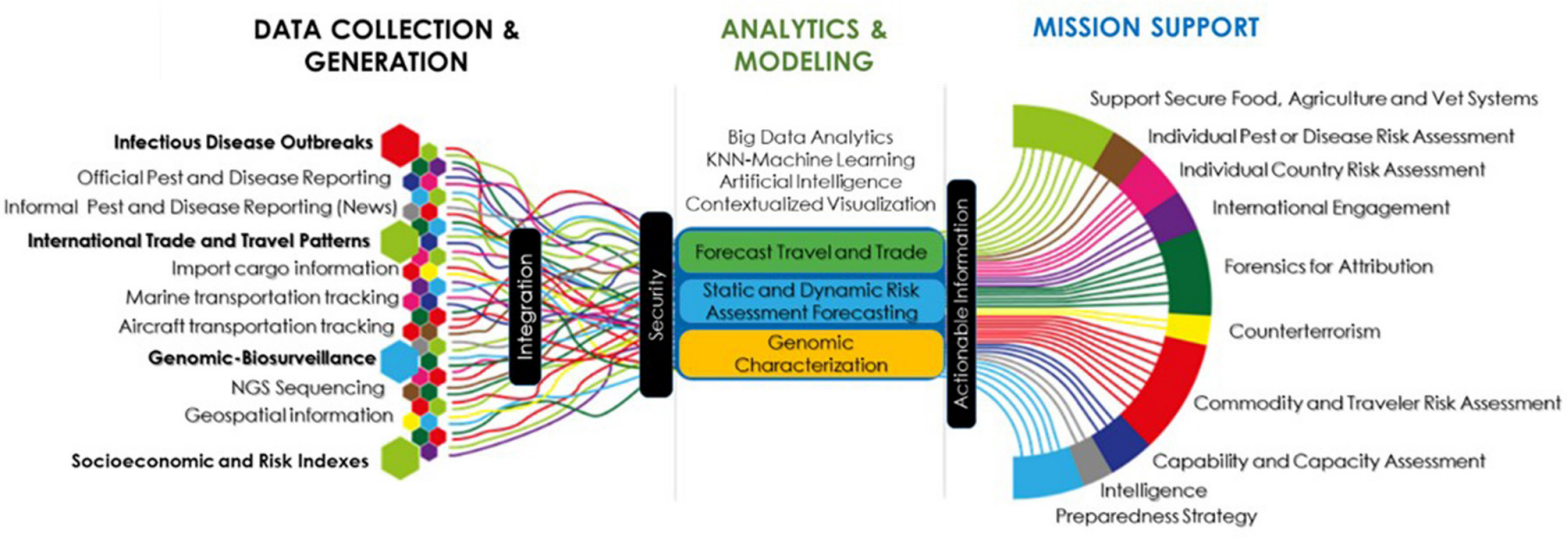
Elements of an autonomous biosurveillance system.

Tracking thousands of infectious agents and toxins, attributing to their origin, identifying proliferation activities of the countries, and detecting outbreaks, is key to quantifying the risk and discriminating hybrid threats. Operating in an increasingly dynamic, complex, and uncertain globalized world imposes new requirements for early warning of infectious agents and toxins affecting humans, animals, and plants. This requires overcoming the limitations of analyst-based biosurveillance systems using autonomous biosurveillance (3). A federated and distributed biosurveillance enterprise should include data collection, integration, disambiguation, analysis, contextualization, and algorithms (Figure 1). This system uses heterogeneous data sources generated at scales of gigabytes per second, including novel or underexplored data sources initially generated not to answer epidemiological questions. Daily passenger arrivals and cargo importations, economic growth rates, buying patterns, trade composition, competitiveness, and dynamics of specific food commodities within different trading partners and nations can complement epidemiological information. Data with different levels of resolution and stakeholders, including but not limited to remote sensing generated by satellites, genomic sequencing, news outlets, aircraft, maritime vessels, and terrestrial cargo movement, are integrated. Disambiguation analysis discriminates misinformation by mapping and scoring reliability and quality using credibility and precision-recall algorithms (55). Because this type and volume of information overwhelm human cognitive capabilities, deep learning analytics and natural language can generate extractive and abstractive summaries from documents with conflicting information (56). These analytical techniques can autonomously access and organize data, translate information from different languages, and reduce human cognitive load and error.

Implementing an early warning pathogen system and a robust autonomous biosurveillance enterprise must avoid a centralized approach for data collection; instead, it should promote a federated multilateral system. The current and future computing power make it possible to analyze increasingly complex information where statistical inferences are limited. This could lead to the construction of different scenarios of the potential impact of specific hybrid threats in human health, agricultural production, and food supply (57). An autonomous biosurveillance enterprise could perform risk assessments using artificial intelligence algorithms that learn, adapt, and evolve as hybrid threats emerge. This process can overcome the cognitive biases that inevitably cloud human judgment and focus on quantitative risk assessments in four-time national security time frames and levels: the immediate and the emerging and the strategic and tactical. More importantly, it can open the possibility for near real-time policymaking assessment and adjustment. Near real-time data-driven analysis tools can provide new insights for evidence-based decision-making. Forecasting tools provide scenarios with probabilities of outcomes and provide some indicators to estimate capability and vulnerability. Such an approach will require a new legislation that improves information exchange efficiency between the authorities, private industry, and other nations. Given the connectivity of agricultural production with the global market and the susceptibility to disruption of the food supply chain, the development of robust autonomous biosurveillance systems requires researchers and public health experts to work closely with personnel in the ports of entry. Such an approach could overcome the limitations of available systems and help policymakers implement and deploy strategic and tactical countermeasures to mitigate the impact of known and unknown pathogens.

## Concluding Remarks

Public health, agricultural production, and food supply chain safety are the backbone of the development of a nation. The disruption of this highly vulnerable system using known and unknown pathogens could trigger severe economic and catastrophic events. This offensive use will remain a feature of the ambiguous and non-traditional hybrid warfare seeking to undermine international norms. Therefore, it is essential for the research and policy community to build new strategies to mitigate the plausible deniability of state and non-state perpetrators. Autonomous biosurveillance integrating data stream and advanced analytics can reduce human cognitive load and error of analyst-based biosurveillance and guide decision-making strategies in near-real-time. This approach can generate new capability enhancements, accelerate international cooperation among public and private stakeholders, and rapidly advance mitigation and resilience strategies countering hybrid threats. We must be prepared.

## Data Availability Statement

The original contributions presented in the study are included in the article/supplementary material, further inquiries can be directed to the corresponding author/s.

## Author Contributions

The author confirms being the sole contributor of this work and has approved it for publication.

## Conflict of Interest

The author declares that the research was conducted in the absence of any commercial or financial relationships that could be construed as a potential conflict of interest.
